# Regulation of expression of Na^+^,K^+^-ATPase in androgen-dependent and androgen-independent prostate cancer

**DOI:** 10.1038/sj.bjc.6690647

**Published:** 1999-09

**Authors:** L J Blok, G T G Chang, M Steenbeek-Slotboom, W M van Weerden, H G P Swarts, J J H H M De Pont, G J van Steenbrugge, A O Brinkmann

**Affiliations:** Departments of Endocrinology & Reproduction and; Experimental Urology, Erasmus University Rotterdam, PO Box 1738, Rotterdam, 3000 DR, The Netherlands; Department of Biochemistry, Institute of Cellular Signalling, PO Box 9101, Nijmegen, 6500 HB, The Netherlands

**Keywords:** Na^+^,K^+^-ATPase, androgens, prostate, androgen-dependent, androgen-independent, cisplatin

## Abstract

The β1-subunit of Na^+^,K^+^-ATPase was isolated and identified as an androgen down-regulated gene. Expression was observed at high levels in androgen-independent as compared to androgen-dependent (responsive) human prostate cancer cell lines and xenografts when grown in the presence of androgens. Down-regulation of the β1-subunit was initiated at concentrations between 0.01 nM and 0.03 nM of the synthetic androgen R1881 after relatively long incubation times (> 24 h). Using polyclonal antibodies, the concentration of β1-subunit protein, but not of the α1-subunit protein, was markedly reduced in androgen-dependent human prostate cancer cells (LNCaP-FGC) cultured in the presence of androgens. In line with these observations it was found that the protein expression of total Na^+^,K^+^-ATPase in the membrane (measured by ^3^H-ouabain binding) was also markedly decreased. The main function of Na^+^,K^+^-ATPase is to maintain sodium and potassium homeostasis in animal cells. The resulting electrochemical gradient is facilitative for transport of several compounds over the cell membrane (for example cisplatin, a chemotherapeutic agent experimentally used in the treatment of hormone-refractory prostate cancer). Here we observed that a ouabain-induced decrease of Na^+^,K^+^-ATPase activity in LNCaP-FGC cells results in reduced sensitivity of these cells to cisplatin-treatment. Surprisingly, androgen-induced decrease of Na^+^,K^+^-ATPase expression, did not result in significant protection against the chemotherapeutic agent. © 1999 Cancer Research Campaign


*British Journal of Cancer* (1999) **81**(1), 28–36
© 1999 Cancer Research Campaign
Article no. bjoc.1999.0647

### Regulation of expression of $Na^+$,$K^+$-ATPase in androgen-dependent and androgen-independent prostate cancer

**LJ Blok[1], GTG Chang[1], M Steenbeek-Slotboom[1], WM van Weerden[2], HGP Swarts[3], JJHHM De Pont[3], GJ van Steenbrugge[2] and AO Brinkmann[1]**

Departments of [1]Endocrinology & Reproduction and [2]Experimental Urology, Erasmus University Rotterdam, PO Box 1738, 3000 DR Rotterdam, The Netherlands; [3]Department of Biochemistry, Institute of Cellular Signalling, University of Nijmegen, PO Box 9101, 6500 HB Nijmegen, The Netherlands

**Summary** The β1-subunit of $Na^+$,$K^+$-ATPase was isolated and identified as an androgen down-regulated gene. Expression was observed at high levels in androgen-independent as compared to androgen-dependent (responsive) human prostate cancer cell lines and xenografts when grown in the presence of androgens. Down-regulation of the β1-subunit was initiated at concentrations between 0.01 nM and 0.03 nM of the synthetic androgen R1881 after relatively long incubation times (> 24 h). Using polyclonal antibodies, the concentration of β1-subunit protein, but not of the α1-subunit protein, was markedly reduced in androgen-dependent human prostate cancer cells (LNCaP-FGC) cultured in the presence of androgens. In line with these observations it was found that the protein expression of total $Na^+$,$K^+$-ATPase in the membrane (measured by $^3$H-ouabain binding) was also markedly decreased. The main function of $Na^+$,$K^+$-ATPase is to maintain sodium and potassium homeostasis in animal cells. The resulting electrochemical gradient is facilitative for transport of several compounds over the cell membrane (for example cisplatin, a chemotherapeutic agent experimentally used in the treatment of hormone-refractory prostate cancer). Here we observed that a ouabain-induced decrease of $Na^+$,$K^+$-ATPase activity in LNCaP-FGC cells results in reduced sensitivity of these cells to cisplatin-treatment. Surprisingly, androgen-induced decrease of $Na^+$,$K^+$-ATPase expression, did not result in significant protection against the chemotherapeutic agent.

**Keywords:** $Na^+$,$K^+$-ATPase; androgens; prostate; androgen-dependent; androgen-independent; cisplatin

$Na^+$,$K^+$-ATPase is a membrane-spanning protein which transports $Na^+$-ions to the outside and $K^+$-ions to the inside of the cell at the expense of ATP, and thus maintains sodium and potassium homeostasis in animal cells (Lingrel and Kuntzweiler, 1994; Rose and Valdes, 1994). $Na^+$,$K^+$-ATPase consists of an α-subunit and a β-subunit present in a 1:1 molar ratio. Up till now, three α- and three β-subunit types have been identified. Different α-subunits can combine with different β-subunits to form bioactive $Na^+$,$K^+$-ATPase. Although the α-subunit is the catalytic subunit of $Na^+$,$K^+$-ATPase, its assembly with a β-subunit in the endoplasmic reticulum is a prerequisite for stable expression, full function and transport to the cell membrane (Geering, 1990; McDonough et al, 1990).

The $Na^+$,$K^+$-ATPase generated electrochemical gradient is responsible for transport of several compounds over the cell membrane. The uptake of cisplatin, a chemotherapeutic agent that has been used in the treatment of hormone-refractory prostate cancer (Akimoto et al, 1994; Coughlin et al, 1994; Sakai et al, 1994; Veronesi et al, 1996), is also regulated in a number of cell systems by $Na^+$,$K^+$-ATPase. Andrews et al. (1991) describe the inhibition of cisplatin uptake by treatment of ovarian carcinoma cells with ouabain, a specific inhibitor of $Na^+$,$K^+$-ATPase activity. Shinohara et al (1994) reported that low levels of $Na^+$,$K^+$-ATPase would protect NIH/3T3 cells against cisplatin-induced apoptosis. Furthermore, a link was made between low levels of expression of $Na^+$,$K^+$-ATPase and insensitivity to cisplatin in non-small-cell lung cancer cells (Ohmori et al, 1994; Kasahara et al, 1996; Bando et al, 1997). Bando et al (1998*a*, 1998*b*), however, reported that in small-cell lung cancer cells this correlation could not be made.

In search for genes that are potentially involved in the transition from hormone-dependent to hormone-independent prostate cancer cell growth, the β1-subunit of $Na^+$,$K^+$-ATPase was identified. In the current study it is shown that expression of the β1-subunit of $Na^+$,$K^+$-ATPase is increased in hormone-independent human prostate cancer xenografts. Furthermore, it is shown that androgens are potent inhibitors of $Na^+$,$K^+$-ATPase activity in androgen-dependent prostate cancer cells.

A problem in the treatment of prostate cancer is that 50% of men, treated for what seemed to be confined prostate cancer, actually already have advanced prostate cancer. Advanced prostate cancer can initially be treated effectively by androgen ablation therapy, but eventually will evolve into androgen-independent prostate cancer. The reason metastatic disease can develop is that the initial treatment of prostate cancer was not effective in removing (surgery) or destroying (irradiation) all prostate cancer cells. At this moment much attention is drawn to develop adjuvant treatment modules to remove cancer cells remaining after the initial treatment (Schnidt et al, 1993; Oliver and Gallagher, 1995; Nelson and Simons, 1996; Chao et al, 1997).

In the current study, investigations were also performed to test the hypothesis that decreased $Na^+$,$K^+$-ATPase expression or

*Received 13 December 1998*
*Revised 15 March 1999*
*Accepted 18 March 1999*

*Correspondence to:* LJ Blok activity in prostate cancer cells would result in reduced sensitivity to cisplatin treatment. The latter investigations may be relevant for improving the effectiveness of treatment of prostate cancer using chemotherapeutic agents.

### MATERIALS AND METHODS

### Cells

The LNCaP-FGC cell line is a gift from Dr JS Horoszewicz (Buffalo, NY, USA) and is identical to the LNCaP cell line which is provided by ATCC (Horoszewicz et al, 1983). For maintenance, these cells were cultured in RPMI-1640 supplemented with 7.5% fetal calf serum (FCS) in the presence of antibiotics. For the experiments, cells were passaged into RPMI-1640 supplemented with 5% stripped serum (dextran-coated charcoal-treated FCS (dcc-FCS)) and were cultured for the indicated times with or without hormones. LNCaP-FGC cells are referred to as androgen-dependent cells, although, in the absence of androgens, these cells will not undergo apoptosis and may even show slow growth (Chang et al, 1997). LNCaP-FGC cells were used between passages 29 and 36. LNCaP-LNO cells (also provided to us by Dr JS Horoszewicz) originate from an early passage of the LNCaP-FGC cells, and grow in RPMI-1640 supplemented with 5% dcc-FCS. LNCaP-LNO cells were used between passages 45 and 50. Concentrations of R1881, vitamin $D_3$, ouabain (Sigma, St Louis, MO, USA) and cisplatin (cis-Platinum(II)diamine Dichloride, Sigma) which were added to the culture media, are indicated in the legends to the Figures.

### Human prostate cancer xenografts

Tumour specimens, obtained from primary carcinomas, metastatic lesions and transurethral resection, were implanted subcutaneously (s.c.) into both shoulders of NMRI male nude mice. All animals had been castrated and supplemented with testosterone before surgery, in order to ensure a similar endocrine background for all tumours. The human prostate cancer xenograft panel used for these investigations contained four androgen-responsive (PC-82, PC-295, PC-310 and PC-346) and five androgen-independent tumours (PC-133, PC-135, PC-324, PC-339 and PC-374). Among the androgen-responsive human prostate cancer xenografts, three xenografts (PC-82, PC-295 and PC-310) are fully dependent on androgens for growth, while one xenograft (PC-346) shows a heterogeneous response when androgens are withdrawn from the tumour-bearing mice. The tumours retained their resemblance to the original patient material, and their main characteristics have been described (van Weerden et al, 1996).

### Differential display PCR

Differential display polymerase chain reaction (ddPCR) was originally performed to detect differences in mRNA expression between the androgen-dependent LNCaP-FGC prostate cancer cell line and the androgen-independent LNCaP-LNO cell line. As outlined in detail by Chang et al (1997), R1881 and vitamin $D_3$ were added in order to obtain similar growth characteristics in both cell lines. LNCaP-FGC and LNCaP-LNO cells were cultured for 6 days in the presence or absence of R1881 (0.1 nM, 10 nM), vitamin $D_3$ (100 nM) or a combination of the two hormones (10 nM R1881 + 100 nM vitamin $D_3$). The biologically active vitamin D metabolite $1,25(OH)_2D_3$ was a gift from Dr L Binderup (Leo Pharmaceutical Products, Ballerup, Denmark) and was donated to us by Dr JPTM van Leeuwen (Department of Internal Medicine, Erasmus University Rotterdam, The Netherlands). Total RNA was isolated and using a random primer (5′-GCAAGCTTGCTA-CAACGAGG-3′ [Pharmacia Biotech, Roosendaal, The Netherlands]) cDNA was generated (MMLV-reverse transcriptase; Life Technologies, Breda, The Netherlands). The cDNA was used in a PCR reaction (Super Taq, Biotechnology Ltd, Cambridge, UK) using the same primer in the forward and reverse reaction in the presence of $^{32}$P-dATP (Amersham, Buckinghamshire, UK). Further details are described by Chang et al (1997).

### RNA isolation and hybridization

Total RNA was isolated and electrophoresed as described by Blok et al (1995). As a probe to detect the β1-subunit of $Na^+$,$K^+$-ATPase, a 191 bp differential display PCR-fragment was used. For PSA mRNA detection, a previously isolated 266 bp differential display PCR-fragment (Blok et al, 1995) was used. The β-actin probe represents a 1.1 kb Pst1-fragment from hamster β-actin.

### Western blot

LNCaP-FGC cells were cultured in RPMI-1640 + 5% dcc-FCS in the presence or absence of 0.1 nM R1881. Subsequently, the cells were lysed and equal amounts of protein were loaded onto a sodium dodecyl sulphate (SDS)-containing polyacrylamide gel, electrophoresed and blotted onto nitrocellulose. A goat polyclonal antibody raised against rabbit α1–β1 $Na^+$,$K^+$-ATPase (Peters et al, 1984; 500-fold diluted) and a peroxidase-conjugated rabbit anti-goat antibody (1:4000 dilution, Sigma) were used to detect $Na^+$,$K^+$-ATPase by chemiluminescense.

### Ouabain binding analysis

LNCaP-FGC cells were cultured to 50% confluence in RPMI-1640 + 5% dcc-FCS in the presence or absence of 0.1 nM R1881. After 3 days the medium was renewed and after 6 days the medium was changed for incubation buffer (40 mM imidazole, pH 7.3, 250 mM sucrose, 5 mM magnesium chloride) containing different amounts of $^3$H-ouabain (2.5 nM, 5 nM, 10 nM, 20 nM, 40 nM, 80 nM) in the presence or absence of 500-fold non-labelled ouabain. $^3$H-ouabain was obtained from Amersham. The cells were incubated with $^3$H-ouabain for 2 h at 37°C. Subsequently, the cells were washed four times with incubation buffer at 0°C, before being lysed in 0.5 M sodium hydroxide (NaOH) for 45 min at 56°C. Samples (50 μl) were counted using liquid scintillation and protein concentrations were measured using Bradford's reagent. Scatchard plots were constructed and $K_d$ and $B_{max}$ values calculated.

### Ouabain/cisplatin incubations

Toxicity of ouabain was measured, after 8 days of culture in a 12-well tissue culture plate in the presence of different concentrations of ouabain, by incubating the cells for 30 min at 37°C with nitro blue tetrazolium (NBT; Sigma) (100 mM phosphate pH 7.4, 0.2 mg $ml^{-1}$ NBT, 0.4 mg $ml^{-1}$ NADH). Mitochondrial diaphorase in cells with ruptured membranes will reduce NBT to $NBTH_2$, a blue-coloured precipitate.

© 1999 Cancer Research Campaign

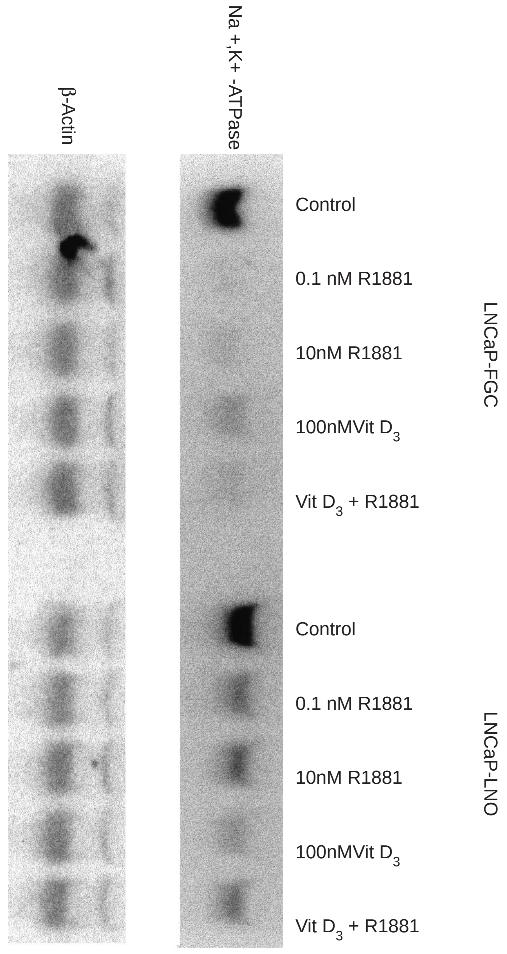

**Figure 1** Expression of the β1-subunit of $Na^+$,$K^+$-ATPase in androgen-dependent LNCaP-FGC and androgen-independent LNCaP-LNO cells. LNCaP-FGC (top 5 lanes) and LNCaP-LNO (bottom 5 lanes) cells were cultured for 6 days in the absence (Control) or presence either of 0.1 nM R1881, 10 nM R1881, 100 nM Vitamin $D_3$ (Vit $D_3$) or 100 nM Vitamin $D_3$ + 10 nM R1881 (Vit $D_3$ + R1881). Cells were harvested and total RNA isolated. Total RNA was loaded on a denaturing gel (20 µg per lane), electrophoresed, blotted and hybridized to the β1-subunit of $Na^+$,$K^+$-ATPase or to hamster β-actin. This experiment has been repeated twice producing similar results

Toxicity of cisplatin was measured after 8 days of culture (which was found to be an optimal time point) of the LNCaP-FGC cells in the presence or absence of different concentrations of cisplatin. Cells were washed once in phosphate-buffered saline (PBS) to remove dead cells and debris. Subsequently, the cells were lysed in 1 M NaOH for 45 min at 56°C and DNA concentrations were measured (Chang et al, 1997). In order to determine whether LNCaP-FGC cell death has characteristics of apoptosis or necrosis when exposed to cisplatin, staining of DNA with Hoechst 33342 and propidium iodide (PI) was performed (Darzynkiewicz et al, 1994). Hoechst 33342 can enter living cells and stain the DNA blue when exposed to 352 nm light. In apoptotic cells the condensed chromatin stains very intense. PI can only stain the DNA of cells with disrupted cellular (plasma and nuclear) membranes. Using this dye, DNA stains red when exposed to 530 nm light. In early apoptotic cells, PI cannot enter the cell, in contrast to late apoptotic cells where the cellular membrane is no longer intact and PI stains the condensed chromatin very intensely. When a cell is necrotic, both Hoechst 33342 and propidium iodide will enter the cell and stain DNA. However, in necrotic cells, the chromatin is not condensed and will only stain diffusely blue and red using Hoechst 33342 and PI respectively (Darzynkiewicz et al, 1994). This method, to make a distinction between apoptotic and necrotic cells after cisplatin treatment, was also used by Lieberthal et al (1996).

### RESULTS

### Differential expression of the β1-subunit of $Na^+$,$K^+$-ATPase

Differential display PCR was performed to identify differences in mRNA expression between the androgen-dependent LNCaP-FGC and the androgen-independent LNCaP-LNO cell line (Chang et al, 1997). Clone 8 was detected as a differentially expressed cDNA between the two cell lines. After cloning the PCR fragment into an appropriate vector, it was established by sequencing that clone 8 was 191 bp in length. When the sequence of clone 8 was compared to known sequences present in the NIH/EMBL databases a 100% homology at the nucleotide level to the β1 subunit of $Na^+$,$K^+$-ATPase was found (fragment 560–751, Ruiz et al, 1995). When mRNA expression of the β1-subunit of $Na^+$,$K^+$-ATPase was analysed on Northern blots in order to verify the ddPCR results, it was observed that under control culture conditions (no hormones added) the expression levels in LNCaP-FGC and LNCaP-LNO cell lines were similar. However, when hormones were added, the β1-subunit of $Na^+$,$K^+$-ATPase was found to be markedly down-regulated by androgens (0.1 nM and 10 nM R1881) and by vitamin $D_3$ (100 nM). Furthermore, androgen-induced down-regulation of mRNA expression of the β1-subunit of $Na^+$,$K^+$-ATPase in the androgen-dependent LNCaP-FGC cells was much more pronounced than in androgen-independent LNCaP-LNO cells (Figure 1).

In human prostate cancer xenografts (grown in testosterone-supplemented castrated nude mice) the expression of the β1-subunit of $Na^+$,$K^+$-ATPase mRNA was low in the four androgen-responsive xenografts (PC-82, PC-295, PC-310 and PC-346). Interestingly, in four out of five androgen-independent xenografts (PC-133, PC-324, PC-339 and PC-374), the expression of β1-subunit mRNA was markedly higher (Figure 2). For unknown reasons, the androgen-independent xenograft PC-135 forms an exception because of its low β1-subunit mRNA expression. As indicated, the xenografts were grown in the presence of testosterone and the findings correlate well with the observed differential expression of the β1-subunit between LNCaP-FGC and LNCaP-LNO cells when these cells were cultured in the presence of androgens (Figure 1, compare LNCaP-FGC lanes 2 and 3 with LNCaP-LNO lanes 2 and 3). A correlation was also observed between the expression of β1-subunit of $Na^+$,$K^+$-ATPase and glandular differentiation of the xenografts; glandular differentiation of the androgen-responsive xenografts varies from well to moderately-well with the expression of the β1-subunit being low, and glandular differentiation of the androgen-independent xenografts ranges from moderate to poor while the expression of the β1-subunit is high (Figure 2). The androgen-responsive PC-346 is an exception having low β1-subunit mRNA expression and poor glandular differentiation.

© 1999 Cancer Research Campaign

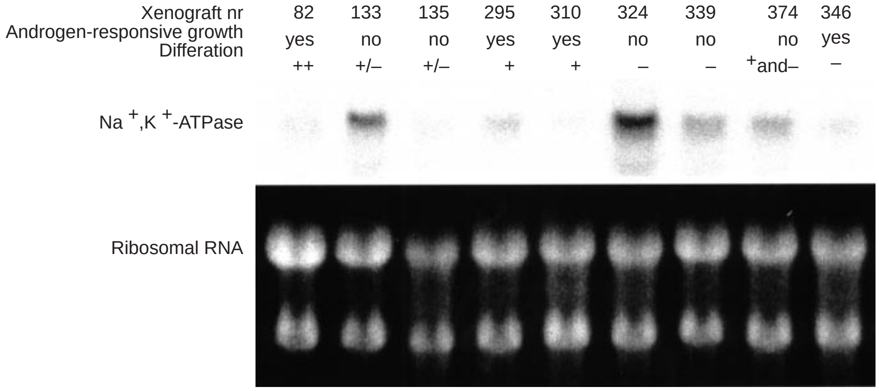

**Figure 2** Expression of the β1-subunit of $Na^+,K^+$-ATPase in androgen-responsive and androgen-independent human prostate cancer xenografts. Xenografts were dissected from mice and immediately frozen in liquid nitrogen. Total RNA was isolated and loaded on a denaturing gel (20 μg per lane), electrophoresed, blotted and hybridized to the β1-subunit of $Na^+,K^+$-ATPase. Ethidium bromide staining of the RNA revealed that there were no differences in loading of the RNA samples. The xenografts were scored for androgen-responsive (yes) or androgen-independent growth (no), and for degree of glandular differentiation (++ = well; + = moderately-well; +/– = moderate; – = poor; + and – = both moderately-well and poorly differentiated glandular tissue in one xenograft) (van Weerden et al, 1996)

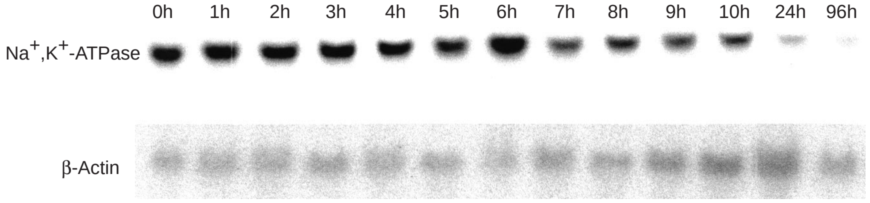

**Figure 3** Time course of androgen-induced down-regulation of β1-subunit of $Na^+,K^+$-ATPase mRNA expression. LNCaP-FGC cells were cultured for 0–96 h in the absence or presence of 0.1 nM R1881. Total RNA, isolated as indicated in Materials and Methods, was loaded on a denaturing gel (20 μg) and electrophoresed, blotted and hybridized to the β1-subunit of $Na^+,K^+$-ATPase or hamster β-actin. This experiment has been repeated three times producing similar results

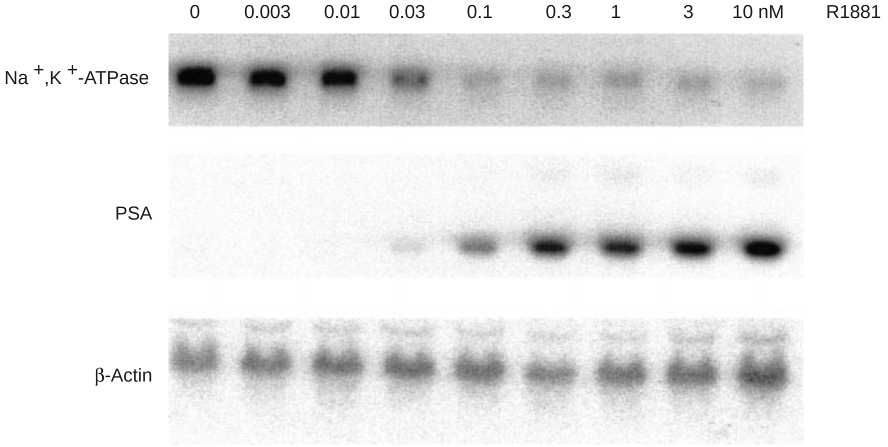

**Figure 4** Regulation of β1-subunit of $Na^+,K^+$-ATPase expression at different androgen concentrations. LNCaP-FGC cells were cultured for 24 h in the absence or presence of 0.003–10 nM R1881. Total RNA, isolated as indicated in Materials and Methods, was loaded on a denaturing gel (20 μg) and electrophoresed, blotted and hybridized to the β1-subunit of $Na^+,K^+$-ATPase, PSA or hamster β-actin. This experiment has been repeated three times producing similar results

### Androgen regulation of $Na^+,K^+$-ATPase expression in LNCaP-FGC cells

Because androgen regulation of the expression of the β1-subunit of $Na^+,K^+$-ATPase was a new finding, this was investigated in more detail. When R1881 (0.1 nM) was added for different time periods to LNCaP-FGC cells in culture, β1 mRNA expression became markedly down-regulated after relatively long incubation times (24 h, Figure 3). Maximal down-regulation of β1-subunit mRNA expression started at R1881 concentrations between 0.01 nM and 0.03 nM (after 24 h). These concentrations are in the same range as the concentrations which are needed for initiation of PSA mRNA up-regulation by the androgen receptor (Murtha et al, 1993) (Figure 4).

© 1999 Cancer Research Campaign 

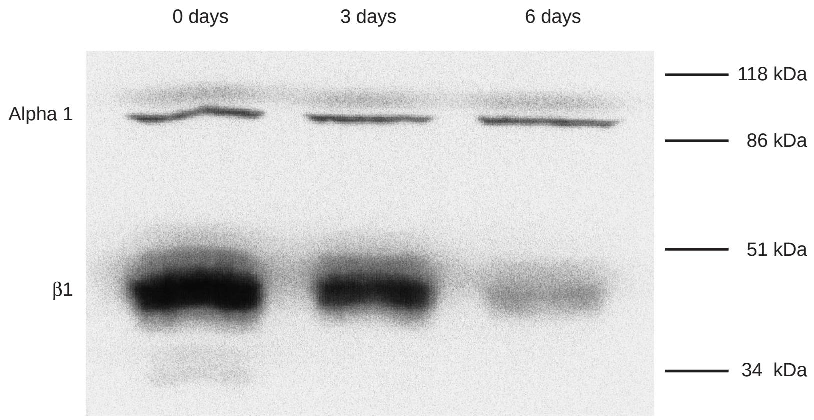

**Figure 5** Androgen regulation of $Na^+$,$K^+$-ATPase protein expression. LNCaP-FGC cells were cultured for 0, 3 or 6 days in the presence of 0.1 nM R1881. The cells were lysed in Laemmli sample buffer, and 5 μg protein was loaded on each lane of the gel, electrophoresed for 1 h at 200 V and blotted. Specific detection of α1- and β1-subunit $Na^+$,$K^+$-ATPase was performed as outlined in the Materials and Methods section. The molecular mass markers are indicated on the right. Due to glycosylation, the β1-subunit appears as a broad protein band between the 51 and 34 kDa markers. This experiment has been repeated twice producing similar results.

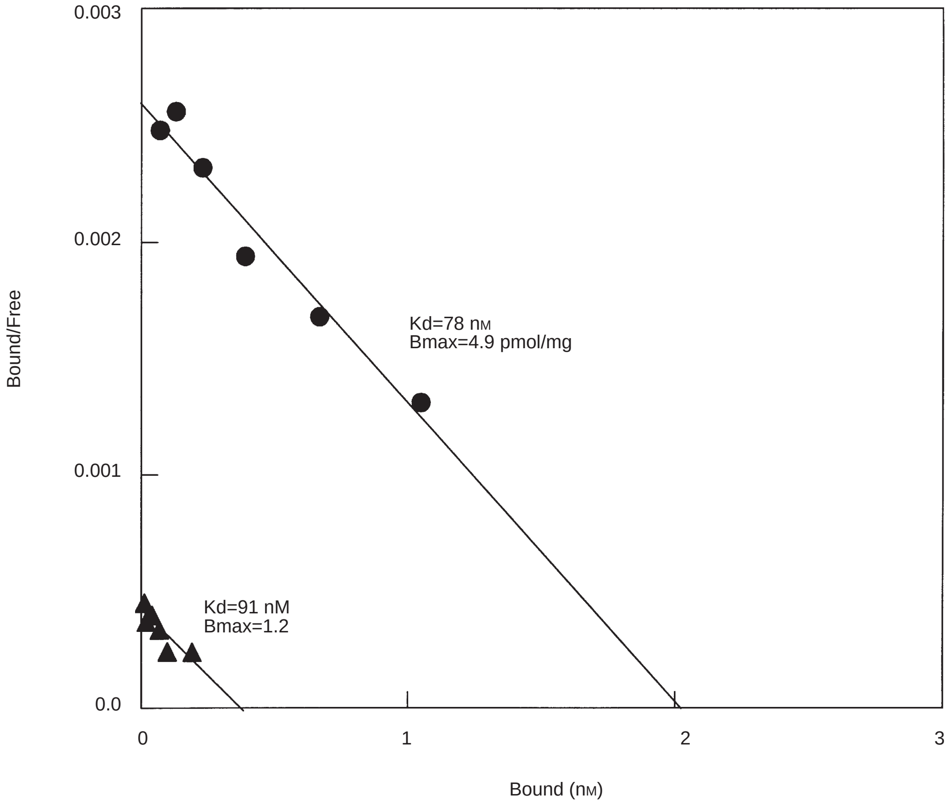

**Figure 6** Androgen regulation of the expression of $Na^+$,$K^+$-ATPase in the membrane. For the Scatchard analysis of $^3$H-ouabain binding to $Na^+$,$K^+$-ATPases expressed in the membrane, LNCaP-FGC cells were cultured to 50% confluence in the presence (▲) or absence (●) of 0.1 nM R1881. After 6 days the medium was replaced for incubation buffer containing $^3$H-ouabain (2.5 nM, 5 nM, 10 nM, 20 nM, 40 nM, 80 nM) in the presence or absence of 500-fold non-labelled ouabain. Cells were incubated for 2 h at 37°C. Subsequently, the cells were harvested and radioactivity was measured. This experiment has been repeated twice producing similar results

In epithelial cells usually the α1- and β1-subunit form the active $Na^+$,$K^+$-ATPase (Lingrel and Kuntzweiler, 1994). Therefore, a goat polyclonal antibody directed against purified rabbit kidney $Na^+$,$K^+$-ATPase was used to detect α1- and β1-subunits of $Na^+$,$K^+$-ATPase in the same cell homogenate. It was observed that the β1-subunit protein was markedly down-regulated in LNCaP-FGC cells after 6 days of culture in the presence of androgens (0.1 nM), while expression of the α1-subunit protein level remained unchanged (Figure 5).

Cardiac glycosides like ouabain cannot enter cells. Their action on $Na^+$,$K^+$-ATPase activity can only occur because these compounds bind with a relatively high specificity to the extracellular domain of the α-subunit which is stabilized in the membrane by the β-subunit (Charlemagne, 1993; Mercer, 1993). Ouabain

© 1999 Cancer Research Campaign

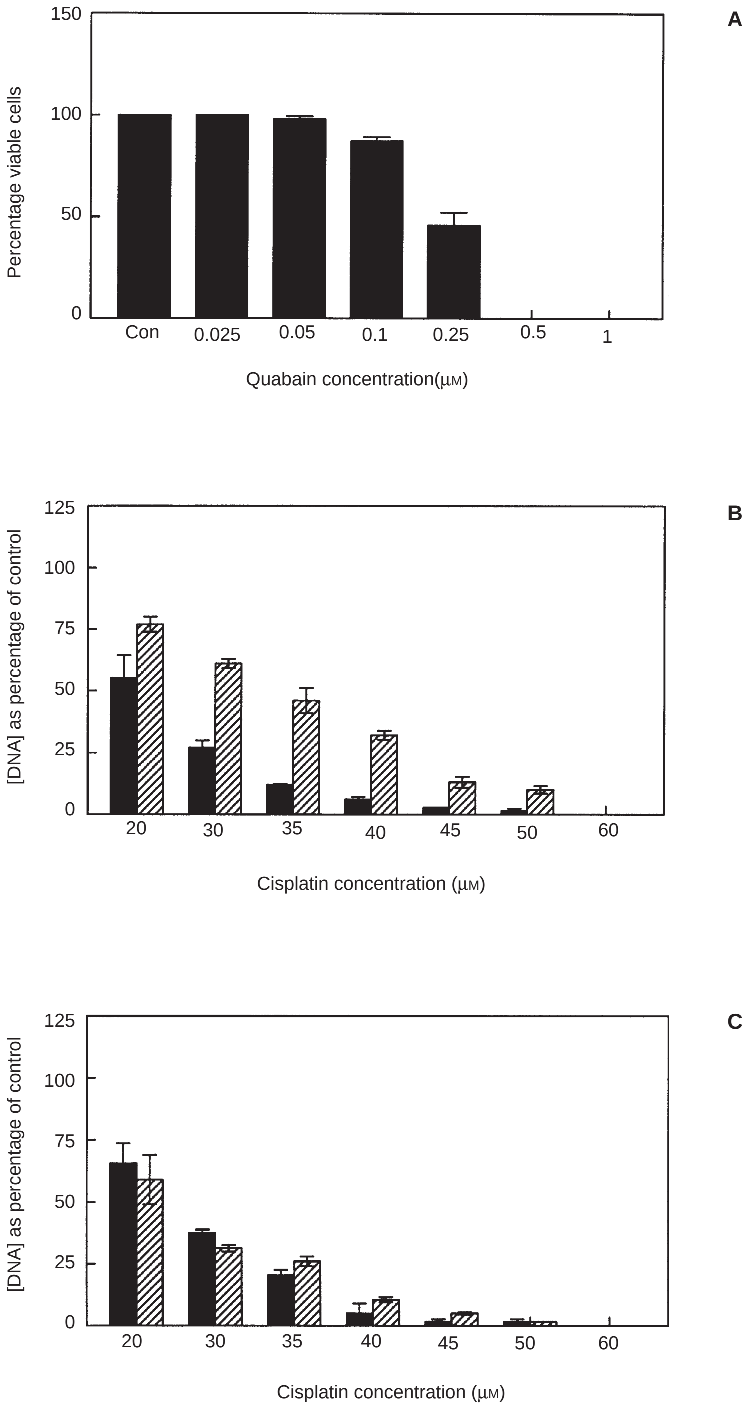

**Figure 7** In contrast to ouabain-induced inhibition of $Na^+,K^+$-ATPase, androgen-induced down-regulation of $Na^+,K^+$-ATPase does not result in protection against cisplatin-induced prostate cancer cell death. (**A**) Cells were cultured for 8 days in the presence of different concentrations (0, 0.025, 0.05, 0.1, 0.25, 0.5 and 1 μM) of the specific inhibitor of $Na^+,K^+$-ATPase, ouabain. Cell viability was measured using the NBT assay, as described in Materials and Methods (panel **A**). (**B**) LNCaP-FGC cells were cultured for 2 h in the absence (black bars) or presence (shaded bars) of 100 nM ouabain before different concentrations cisplatin were added to the culture medium (0, 20, 30, 35, 40, 45, 50 and 60 μM). After 8 days the cells were harvested and DNA measured as indicated in the Materials and Methods section (panel **B**). (**C**) LNCaP-FGC cells were cultured for 6 days in the absence (black bars) or presence (shaded bars) of 0.1 nM R1881. Subsequently different concentrations cisplatin were added to the culture medium (0, 20, 30, 35, 40, 45, 50 and 60 μM), and culture was continued for 8 days. Cells were harvested and DNA was measured as indicated in the Materials and Methods section (panel **C**). These experiments were repeated at least twice producing similar results binding will result in decreased activity of $Na^+,K^+$-ATPase. However, radioactively labelled ouabain can also be used to measure the amount of $Na^+,K^+$-ATPase expressed in the membrane. In order to do so in control or R1881 pretreated (6 days) LNCaP-FGC cells, these cells were incubated with different concentrations of $^3H$-ouabain in the presence or absence

*© 1999 Cancer Research Campaign* 

of 500-fold non-labelled ouabain for a short period of time (Figure 6). Scatchard analysis on living cells revealed a $K_d$ of 78 nM in the control situation and 91 nM in case of R1881 pretreatment. The $B_{max}$ value of control cells was 4.9 pmol mg$^{-1}$ protein and about fourfold higher than the $B_{max}$ of R1881 pretreated cells (1.2 pmol mg$^{-1}$ protein). These data are in line with the observations described in Figure 5, and indicate that androgen-induced down-regulation of the β1-subunit protein expression is rate-limiting for the expression of the α,β-heterodimer of $Na^+$,$K^+$-ATPase at the cell surface (as measured by $^3$H-ouabain binding).

### Inhibition of cisplatin-induced cell death

Cisplatin induces cell death in most culture systems. It was observed that cisplatin induces apoptosis at 40 μM and 70 μM and induces necrosis at much higher concentrations (500 μM; data not shown). In all further experiments we have used cisplatin at concentrations below 70 μM.

Cisplatin accumulation in the cell is partly dependent on the $K^+$ influx; when the $K^+$ gradient is steep, cisplatin will enter the cell relatively easy (Andrews et al, 1991). The $K^+$ gradient is generated by $Na^+$,$K^+$-ATPase, and there are several indications that reduced expression or activity of $Na^+$,$K^+$-ATPase will result in reduced uptake of cisplatin (Andrews et al, 1991; Shinohara et al, 1994; Ohmori et al, 1994; Kasahara et al, 1996; Bando et al, 1997, 1998*a*, 1998*b*). For prostate cancer the relation between expression or activity of $Na^+$,$K^+$-ATPase and effectiveness of cisplatin treatment has not been investigated to this point.

One way to inhibit $Na^+$,$K^+$-ATPase activity specifically is to culture LNCaP-FGC cells in the presence of ouabain. However, the concentration of ouabain should be low because otherwise the $Na^+$/$K^+$ homeostasis becomes too severely disrupted and the cells will die. In order to investigate which concentration of ouabain could be used, LNCaP-FGC cells were cultured in the presence of different concentrations of ouabain and cell viability was measured. It was observed that a concentration of 100 nM ouabain would only marginally reduce viability of the cells (Figure 7A).

Next, it was observed that LNCaP-FGC cells cultured in the presence of 100 nM ouabain were much more resistant to cisplatin-induced apoptosis than control cells at cisplatin concentrations of 20–50 μM (Figure 7B). The next step was to culture LNCaP-FGC cells in the presence of 0.1 nM R1881 for 6 days ($Na^+$,$K^+$-ATPase expression becomes reduced), followed by treatment with different concentrations of cisplatin for 1 week. In contrast to the decrease in $Na^+$,$K^+$-ATPase activity by ouabain, androgen-induced down-regulation of expression of $Na^+$,$K^+$-ATPase did not result in a clear protection against cisplatin-induced prostate cancer cell death. This suggests that potentially other androgen-induced import-mechanisms are counteracting the effect of androgen-induced down-regulation of $Na^+$,$K^+$-ATPase on cisplatin-induced cell death.

### DISCUSSION

### Regulation of expression of $Na^+$,$K^+$-ATPase in prostate cancer

$Na^+$,$K^+$-ATPase is necessary for the maintenance of sodium and potassium homeostasis in eukaryotic cells. Regulation of $Na^+$,$K^+$-ATPase activity can be achieved by various routes: intracellular $Na^+$ concentration is an important regulator, the abundance of the subunits can be controlled by transcriptional or post-transcriptional regulation (aldosterone in kidney and thyroid hormone in myocardium) (Hensley et al, 1992; Wheling et al, 1993; Ewart and Klip, 1995; Middleton, 1996), but it is also possible to regulate activity of the α-subunit by binding of inhibitors as ouabain and bufalin (MacGregor and Walker, 1993).

Recently, the promoter of the β1-subunit of $Na^+$,$K^+$-ATPase was shown to contain several mineralocorticoid and glucocorticoid responsive elements (MRE/GRE) (Derfoul et al, 1998). When a large portion of the promoter region (–1141 to +490) was cloned in front of a luciferase reporter gene, profound up-regulation of transcriptional activity was found in the presence of glucocorticoid receptors in CV-1 cells. It is true that androgen receptors and glucocorticoid receptors can bind to similar responsive elements on DNA (Van Dijck et al, 1989). However, there are also distinct differences between the two receptors which contribute to receptor-selective transcriptional regulation (Rundlett and Miesfeld, 1995; Claessens et al, 1996; Scheller et al, 1998). It is interesting that, in contrast to the findings of Derfoul et al (1998), in the current investigations androgens are potent inhibitors of the expression of the β1-subunit of $Na^+$,$K^+$-ATPase in LNCaP cells. At the basis of the difference in regulation of β1-subunit mRNA expression by glucocorticoid and androgen receptors may be distinct receptor differences, but promoter and cell context (CV-1 vs LNCaP) may also play an important role.

Down-regulation of the β1-subunit of $Na^+$,$K^+$-ATPase is much more pronounced in the androgen-dependent LNCaP-FGC cells than in androgen-independent LNCaP-LNO cells. In other words, the β1-subunit of $Na^+$, $K^+$-ATPase is expressed at higher levels in androgen-independent LNCaP-LNO cells cultured in the presence of androgens than in androgen-dependent LNCaP-FGC cells cultured under similar conditions. This observation was also done in human prostate cancer xenografts: androgen-independent xenografts express higher levels of the β1-subunit of $Na^+$,$K^+$-ATPase than androgen-responsive xenografts when grown in the presence of testosterone. The only exception is xenograft PC-135 which seems to express low levels of β1-subunit mRNA. Because of these findings it may be of interest to investigate whether gain of expression of the β1-subunit of $Na^+$,$K^+$-ATPase can be used as a molecular marker to distinguish between androgen-dependent and androgen-independent prostate cancer cells in biopsy material from patients who have not been treated by androgen ablation therapy. Another point of interest is that chromosomal localization of the β1-subunit of $Na^+$,$K^+$-ATPase on 1q24–25 is in the same region as the putative prostate cancer-susceptibility locus HPC1 (Cooney et al, 1997).

### Inhibition of cisplatin-induced cell death

Cisplatin is a chemotherapeutic agent that has been used experimentally as adjuvant therapy in the treatment of hormone refractory prostate cancer; the effectiveness of this treatment has always been limited (Akimoto et al, 1994; Coughlin et al, 1994; Sakai et al, 1994; Veronesi et al, 1996). In the current investigation it is shown that the chemotherapeutic effectiveness of cisplatin treatment of a prostate cancer cell line is affected by $Na^+$,$K^+$-ATPase activity. When $Na^+$,$K^+$-ATPase activity is reduced using ouabain as a specific inhibitor, the sensitivity to cisplatin-induced cell death is reduced. Surprisingly, when the prostate cancer cells

© 1999 Cancer Research Campaign were treated with androgens, which reduces the amount of $Na^+,K^+$-ATPase, cisplatin-induced cell death was only marginally affected. An explanation could be that in LNCaP-FGC cells other androgen-regulated mechanisms are counteracting the effect of reduced $Na^+,K^+$-ATPase expression on cisplatin-induced apoptosis. In small-cell lung cancer cells the importance of $Na^+,K^+$-ATPase as an active transporter of cisplatin was also found to be limited, indicating that also in these cells other mechanisms of cisplatin transport are operational (Bando et al, 1998*a*, 1998*b*).

Thus far, cisplatin treatment of hormone refractory prostate cancer patients has not been very effective. Here we show that down-regulation of $Na^+,K^+$-ATPase can, under certain circumstances, further reduce the effectiveness of cisplatin treatment of prostate cancer cells. If, however, a method could be developed to increase $Na^+,K^+$-ATPase expression in prostate cancer cells, it should, at least in theory, be possible to increase the effectiveness of cisplatin as adjuvant treatment against hormone refractory prostate cancer.

### ACKNOWLEDGEMENTS

This work was supported in part by a grant from the Dutch Cancer Society (EUR 95-1031), by a grant from The Netherlands Organisation for Scientific Research (903-46-169) and by the Erasmus Trust Fund.

### REFERENCES

Akimoto S, Ohki T, Akakura K, Masai M and Shimazaki J (1994) Chemotherapy for endocrine-therapy-refractory prostate cancer. *Cancer Chemother Pharmacol* **35**: S18–22

Andrews PA, Mann SC, Huynh H and Albright KD (1991) Role of the $Na^+,K^+$-adenosine triphosphatase in the accumulation of *cis*-diamminedichloroplatinum(II) in human ovarian carcinoma cells. *Cancer Res* **51**: 3677–3681

Bando T, Fujimura M, Kasahara K, Shibata K, Heki U, Iwasa K, Ueda A, Tomikawa S and Matsuda T (1997) Exposure to sorbitol induces resistance to cisplatin in human non-small-cell lung cancer cell lines. *Anticancer Res* **17**: 3345–3348

Bando T, Fuijmura M, Kasahara K and Matsuda T (1998*a*) Significance of $Na^+,K^+$-ATPase on intracellular accumulation of cis-diamminedichlorideplatinum(II) in human non-small-cell but not in small-cell lung cancer cell lines. *Anticancer Res* **18**: 1085–1089

Bando T, Fujimura M, Kasahara K and Matsuda T (1998*b*) Role of thromboxane receptor on the intracellular accumulation of cis-diamminedichlorideplatinum(II) in non-small-cell but not in small-cell lung cancer cell lines. *Anticancer Res* **18**: 1079–1084

Blok LJ, Kumar MV and Tindall DJ (1995) Isolation of cDNAs that are differentially expressed between androgen-dependent and androgen-independent prostate carcinoma cells using differential display PCR. *Prostate* **26**: 213–224

Blok LJ, De Ruiter PE and Brinkmann AO (1998) Forskolin-induced dephosphorylation of the androgen receptor impairs ligand binding. *Biochemistry* **37**: 3850–3857

Chang GTG, Blok LJ, Steenbeek M, Veldscholte J, van Weerden WM, van Steenbrugge GJ and Brinkmann AO (1997) Differentially expressed genes in androgen-dependent and androgen-independent prostate carcinomas. *Cancer Res* **57**: 4075–4081

Chao D, von Schlippe M and Harland SJ (1997) A phase II study of continuous infusion 5-fluorouracil (5-FU) with epirubicin and cisplatin in metastatic, hormone-resistant prostate cancer: an active new regime. *Eur J Cancer* **33**: 1230–1233

Charlemagne D (1993) Molecular and cellular levels of action of digitalis. *Herz* **18**: 79–85

Claessens F, Alen P, Devos A, Peeters B, Verhoeven G and Rombauts W (1996) The androgen-specific probasin response element 2 interacts differentially with androgen and glucocorticoid receptors. *J Biol Chem* **271**: 19013–19016

Cooney KA, McCarthy JD, Lange E, Huang L, Miesfeldt S, Montie JE, Oesterling JE, Sandler HM and Lange K (1997) Prostate cancer susceptibility locus on chromosome 1q: a confirmatory study. *J Natl Cancer Inst* **89**: 955–959

Coughlin CT, Richmond RC and Page RL (1994) Platinum drug delivery and radiation for locally advanced prostate cancer. *Int J Radiation Oncol Biol Phys* **28**: 1029–1038

Darzynkiewicz Z, Li X and Gong J (1994) Assays of cell viability: discrimination of cells dying by apoptosis. *Methods Cell Biol* **41**: 15–39

Derfoul A, Robertson NM, Lingrel JB, Hall DJ and Litwack G (1998) Regulation of the human Na/K-ATPase β1 gene promoter by mineralocorticoid and glucocorticoid receptors. *J Biol Chem* **273**: 20702–20711

Ewart HS and Klip A (1995) Hormonal regulation of the $Na^+,K^+$-ATPase: mechanisms underlying rapid and sustained changes in pump activity. *Am J Physiol* **38**: C295–311

Geering K (1990) Subunit assembly and functional maturation of $Na^+,K^+$-ATPase. *J Membr Biol* **115**: 109–121

Hensley CB, Azuma KK, Tang M-J and McDonough AA (1992) Thyroid hormone induction of rat myocardial $Na^+$-$K^+$-ATPase: α1-, α2-, and β1-mRNA and -protein levels at steady state. *Am J Physiol* **262**: C484–492

Horoszewicz JS, Leong SS, Kawinski E, Karr JP, Rosenthal H, Chu TM, Mirand EA and Murphy GP (1983) LNCaP model of human prostatic carcinoma. *Cancer Res* **43**: 1809–1818

Kasahara K, Fujimura M, Bando T, Shibata K and Matsuda T (1996) Modulation of sensitivity to cis-diamminedichloroplatinum(II) by tromboxane $A_2$ receptor antagonists in non-small-cell lung cancer cell lines. *Br J Cancer* **74**: 1553–1558

Lieberthal W, Triaca V and Levine J (1996) Mechanisms of death induced by cisplatin in proximal tubular epithelial cells: apoptosis vs. necrosis. *Am J Physiol* **270**: F700–708

Lingrel JB and Kuntzweiler T (1994) $Na^+,K^+$-ATPase. *J Biol Chem* **269**: 19659–19662

MacGregor SE and Walker JM (1993) Inhibitors of the $Na^+,K^+$-ATPase. *Comp Biochem Physiol* **105C**: 1–9

McDonough AA, Geering K and Farley RA (1990) The sodium pump needs its β subunit. *FASEB J* **4**: 1598–1605

Mercer RW (1993) Structure of the Na,K-ATPase. *Int Rev Cytol* **137C**: 139–168

Middleton JP (1996) Direct regulation of the Na,K pump by signal transduction mechanisms. *Miner Electrolyte Metab* **22**: 293–302

Murtha P, Tindall DJ and Young CY (1993) Androgen induction of a human prostate-specific kalliklein, hKLK2: characterization of an androgen response element in the 5´promoter region of the gene. *Biochemistry* **32**: 6459–6464

Nelson WG and Simons W (1996) New approaches to adjuvant therapy for patients with adverse histopathologic findings following radical prostatectomy. *Urol Clin North Am* **23**: 685–696

Ohmori T, Nishio K, Ohta S, Kubota N, Adachi M, Komiya K and Saijo N (1994) Ouabain-resistant non-small-cell lung-cancer cell line shows collateral sensitivity to cis-diamminedichloroplatinum(II) (CDDP). *Int J Cancer* **57**: 111–116

Oliver RTD and Gallagher CJG (1995) Intermittent endocrine therapy and its potential for chemoprevention of prostate cancer. *Cancer Surv* **23**: 191–207

Peters WHM, Ederveen AGH, Salden MHL, De Pont JJHHM and Bonting SL (1984) Lack of immunological cross reactivity between transport enzymes ($Na^+$+$K^+$)-ATPase and ($K^+$+$H^+$)-ATPase. *J Bioenerg Biomembr* **16**: 223–232

Rose AM and Valdes R (1994) Understanding the sodium pump and its relevance to disease. *Clin Chem* **40**: 1674–1685

Ruiz A, Bhat SP and Bok D (1995) Characterization and quantitation of full length and truncated Na,K-ATPase α1 and β1 RNA transcripts expressed in human retinal pigment epithelium. *Genes* **155**: 179–184

Rundlett SE and Miesfeld RL (1995) Quantitative differences in androgen and glucocorticoid receptor DNA binding properties contribute to receptor-selective transcriptional regulation. *Mol Cell Endocrinol* **109**: 1–10

Sakai H, Minami Y, Kanetake H and Saito Y (1994) Chemo-endocrine therapy for prostate cancer with bone metastasis. *Cancer Chemother Pharmacol* **35**: S23–26

Scheller A, Hughes E, Golden KL and Robins DM (1998) Multiple receptor domains interact to permit, or restrict, androgen-specific gene activation. *J Biol Chem* **273**: 24216–24222

Schnidt JD, Gibbons RP, Murphy GP and Bartolucci A (1993) Adjuvant therapy for localized prostate cancer. *Cancer* **71**: 1005–1013

Shinohara N, Ogiso Y, Arai T, Takami S, Nonomura K, Koyanagi T and Kuzumaki N (1994) Differential $Na^+,K^+$-ATPase activity and cisplatin sensitivity between transformants induced by H-ras and those induced by K-*ras*. *Int J Cancer* **58**: 672–677

van Dijck P, Winderickx J, Heyns W and Verhoeven G (1989) Binding of androgen–receptor complexes to alpha 2u-globulin genes and to the long terminal repeat of mouse mammary tumor virus. *Mol Cell Endocrinol* **64**: 195–204

van Weerden WM, de Ridder CMA, Verdaasdonk CL, Romijn JC, van der Kwast TH, Schroder FH and van Steenbrugge GJ (1996) Seven newly developed human prostate tumor xenograft models: establishment and histopathological characterization. *Am J Pathol* **149**: 1055–1062

Veronesi A, Re GL, Foladore S, Merlo A, Giuliotto N, Talamini R and Monfardini S (1996) Multidrug chemotherapy in the treatment of non-elderly patients with hormone-refractory prostatic carcinoma. A phase II study. North-Eastern Italian Oncology Group (GOCCNE). *Eur Urol* **29**: 434–438

Wehling M, Christ M and Gerzer R (1993) Aldosterone-specific membrane receptors and related rapid, non-genomic effects. *Trends Pharmacol Sci* **14**: 1–3

 *© 1999 Cancer Research Campaign*

## References

[PDF_00798] Andrews P. A., Mann S. C., Huynh H. H., Albright K. D. (1991). Role of the Na+, K(+)-adenosine triphosphatase in the accumulation of cis-diamminedichloroplatinum(II) in human ovarian carcinoma cells.. Cancer Res.

[PDF_00813] Bando T., Fujimura M., Kasahara K., Matsuda T. (1998). Role of thromboxane receptor on the intracellular accumulation of cis-diamminedichloroplatinum(II) in non-small-cell but not in small-cell lung cancer cell lines.. Anticancer Res.

[PDF_00807] Bando T., Fujimura M., Kasahara K., Matsuda T. (1998). Significance of Na+, K(+)-ATPase on intracellular accumulation of cis-diamminedichloroplatinum(II) in human non-small-cell but not in small-cell lung cancer cell lines.. Anticancer Res.

[PDF_00804] Bando T., Fujimura M., Kasahara K., Shibata K., Shirasaki H., Heki U., Iwasa K., Ueda A., Tomikawa S., Matsuda T. (1997). Exposure to sorbitol induces resistance to cisplatin in human non-small-cell lung cancer cell lines.. Anticancer Res.

[PDF_00817] Blok L. J., Kumar M. V., Tindall D. J. (1995). Isolation of cDNAs that are differentially expressed between androgen-dependent and androgen-independent prostate carcinoma cells using differential display PCR.. Prostate.

[PDF_00821] Blok L. J., de Ruiter P. E., Brinkmann A. O. (1998). Forskolin-induced dephosphorylation of the androgen receptor impairs ligand binding.. Biochemistry.

[PDF_00824] Chang G. T., Blok L. J., Steenbeek M., Veldscholte J., van Weerden W. M., van Steenbrugge G. J., Brinkmann A. O. (1997). Differentially expressed genes in androgen-dependent and -independent prostate carcinomas.. Cancer Res.

[PDF_00828] Chao D., von Schlippe M., Harland S. J. (1997). A phase II study of continuous infusion 5-fluorouracil (5-FU) with epirubicin and cisplatin in metastatic, hormone-resistant prostate cancer: an active new regimen.. Eur J Cancer.

[PDF_00837] Cooney K. A., McCarthy J. D., Lange E., Huang L., Miesfeldt S., Montie J. E., Oesterling J. E., Sandler H. M., Lange K. (1997). Prostate cancer susceptibility locus on chromosome 1q: a confirmatory study.. J Natl Cancer Inst.

[PDF_00853] Geering K. (1990). Subunit assembly and functional maturation of Na,K-ATPase.. J Membr Biol.

[PDF_00862] Horoszewicz J. S., Leong S. S., Kawinski E., Karr J. P., Rosenthal H., Chu T. M., Mirand E. A., Murphy G. P. (1983). LNCaP model of human prostatic carcinoma.. Cancer Res.

[PDF_00865] Kasahara K., Fujimura M., Bando T., Shibata K., Shirasaki H., Matsuda T. (1996). Modulation of sensitivity to cis-diamminedichloroplatinum (II) by thromboxane A2 receptor antagonists in non-small-cell lung cancer cell lines.. Br J Cancer.

[PDF_00880] McDonough A. A., Geering K., Farley R. A. (1990). The sodium pump needs its beta subunit.. FASEB J.

[PDF_00882] Mercer R. W. (1993). Structure of the Na,K-ATPase.. Int Rev Cytol.

[PDF_00883] Middleton J. P. (1996). Direct regulation of the Na,K pump by signal transduction mechanisms.. Miner Electrolyte Metab.

[PDF_00885] Murtha P., Tindall D. J., Young C. Y. (1993). Androgen induction of a human prostate-specific kallikrein, hKLK2: characterization of an androgen response element in the 5' promoter region of the gene.. Biochemistry.

[PDF_00888] Nelson W. G., Simons J. W. (1996). New approaches to adjuvant therapy for patients with adverse histopathologic findings following radical prostatectomy.. Urol Clin North Am.

[PDF_00891] Ohmori T., Nishio K., Ohta S., Kubota N., Adachi M., Komiya K., Saijo N. (1994). Ouabain-resistant non-small-cell lung-cancer cell line shows collateral sensitivity to cis-diamminedichloroplatinum(II) (CDDP).. Int J Cancer.

[PDF_00895] Oliver R. T., Gallagher C. J. (1995). Intermittent endocrine therapy and its potential for chemoprevention of prostate cancer.. Cancer Surv.

[PDF_00897] Peters W. H., Ederveen A. G., Salden M. H., de Pont J. J., Bonting S. L. (1984). Lack of immunological cross reactivity between the transport enzymes (Na+ + K+)-ATPase and (K+ + H+)-ATPase.. J Bioenerg Biomembr.

[PDF_00904] Rose A. M., Valdes R. (1994). Understanding the sodium pump and its relevance to disease.. Clin Chem.

[PDF_00918] Schmidt J. D., Gibbons R. P., Murphy G. P., Bartolucci A. (1993). Adjuvant therapy for localized prostate cancer.. Cancer.

[PDF_00944] Schoepp D. D., Conn P. J. (1993). Metabotropic glutamate receptors in brain function and pathology.. Trends Pharmacol Sci.

[PDF_00920] Shinohara N., Ogiso Y., Arai T., Takami S., Nonomura K., Koyanagi T., Kuzumaki N. (1994). Differential Na+,K(+)-ATPase activity and cisplatin sensitivity between transformants induced by H-ras and those induced by K-ras.. Int J Cancer.

[PDF_00940] Veronesi A., Re G. L., Foladore S., Merlo A., Giuliotto N., Talamini R., Monfardini S. (1996). Multidrug chemotherapy in the treatment of non-elderly patients with hormone-refractory prostatic carcinoma. A phase II study. North-Eastern Italian Oncology Group (GOCCNE).. Eur Urol.

[PDF_00936] van Weerden W. M., de Ridder C. M., Verdaasdonk C. L., Romijn J. C., van der Kwast T. H., Schröder F. H., van Steenbrugge G. J. (1996). Development of seven new human prostate tumor xenograft models and their histopathological characterization.. Am J Pathol.

